# Association of Cumulative Proton Pump Inhibitor Use with Prostate Cancer Risk and Outcomes: A Population-Based Cohort Study

**DOI:** 10.1158/2767-9764.CRC-26-0098

**Published:** 2026-07-24

**Authors:** Rashid K. Sayyid, Raj Tiwari, Bo Zhang, Andrew S. Wilton, Katherine Lajkosz, Jessica Grace Cockburn, Rui M. Bernardino, Zizo Al-Daqqaq, Linda Z. Penn, Hanan Goldberg, Refik Saskin, Neil E. Fleshner

**Affiliations:** 1 Department of Urology, The University of Arizona, Banner University Medical Center, Tucson, Arizona.; 2 Department of Urology, Sengkang General Hospital, Singapore, Singapore.; 3 ICES, Toronto, Canada.; 4 Department of Biostatistics, Princess Margaret Cancer Centre, Toronto, Canada.; 5 Department of Surgical Oncology, Princess Margaret Cancer Centre, Toronto, Canada.; 6 Division of Urology, Department of Surgical Oncology, University of Toronto, Toronto, Canada.; 7 Division of Urology, Department of Surgery, The Ottawa Hospital, Ottawa, Canada.; 8 Princess Margaret Cancer Centre, Toronto, Canada.; 9 Department of Medical Biophysics, University of Toronto, Toronto, Canada.; 10 Department of Urology, SUNY Upstate Medical University, Syracuse, New York.; 11 Institute of Health Policy, Management, and Evaluation, University of Toronto, Toronto, Canada.

## Abstract

Proton pump inhibitor (PPI) use has been associated with increased prostate cancer risk in case–control and cohort studies of men with a negative prostate biopsy. We estimated the association of cumulative PPI use with prostate cancer diagnosis and outcomes in a biopsy-naïve cohort of men using provincewide-linked administrative data from Ontario, Canada, including men ≥66 years with no prior prostate cancer diagnosis, biopsy, or treatment and no PPI/histamine-2–blocker prescriptions within the year preceding study inclusion (January 2003–December 2018). The primary outcome was time to prostate cancer diagnosis. Associations were evaluated using univariable/multivariable logistic regression models with complementary log–log modeling. Median follow-up was 9.2 years (*n* = 559,425), and 30.2% had any PPI use during follow-up. Annual PPI use increased from 2.4% (2003) to 13.8% (2019). Cumulative PPI use was associated with lower prostate cancer diagnosis rates [hazard ratio (HR) for highest user quintile vs. nonusers: 0.75, 95% confidence interval (CI), 0.71–0.79]. Following adjustment for the frequency of general practitioner visits and prostate-specific antigen tests in the preceding 2 years, PPI use was no longer associated with prostate cancer diagnosis (HR, 0.99; 95% CI, 0.93–1.05). No association was observed between PPI use and rates of clinically significant or high-grade prostate cancer (HRs, 0.99 and 1.04, respectively). Cumulative PPI use was associated with up to 17% lower rates of a first androgen deprivation therapy prescription or bilateral orchiectomy. Cumulative PPI use was not associated with prostate cancer diagnosis rates, including clinically significant and high-grade prostate cancer. These findings do not support an independent association between PPI use and prostate cancer risk.

**Significance:**

Among biopsy-naïve older men, cumulative PPI use was not associated with prostate cancer diagnosis, including clinically significant and high-grade disease, after adjusting for healthcare resource utilization. These findings challenge prior observational signals suggesting increased risk and indicate that detection bias likely explains earlier associations.

## Introduction

Given the increased incidence of prostate cancer in the developed world (1), there has been long-standing interest in identifying modifiable risk factors for primary and/or secondary prevention (2–4). Proton pump inhibitors (PPI), prescribed for gastrointestinal disorders, including gastroesophageal reflux disease (GERD), inhibit gastric acid secretion by irreversibly binding and inhibiting gastric parietal cell hydrogen/potassium ATPase (5). PPIs are approved for short-term use (<8 weeks); however, population-level data demonstrate that long-term, off-label use is increasing, with 6% to 10% of Americans currently using PPIs long-term (6, 7). Chronic PPI use has been associated with adverse events, including osteoporotic fractures, chronic kidney disease, and serious infections (8). Initially thought to have potential antitumor effects via regulation of cancer cell apoptosis, metastasis, and autophagy (9, 10), recent evidence suggests that long-term PPI use may be associated with increased risks of gastric, hepatic, and esophageal malignancies (11–13). *In vitro* prostate cancer studies have demonstrated that PPIs promote tumor growth in mice via increased cell survival/proliferation and inhibition of apoptosis in LNCaP cells, with cell-cycle progression mediated via increased c-Myc expression, ErbB2 activity, and prostate-specific antigen (PSA) secretion (14).

Previous studies suggest that PPI use may be associated with increased prostate cancer risk. An Icelandic population-based case–control study demonstrated that “ever” PPIs use was associated with 12% increased odds of a prostate cancer diagnosis (15), and a select population-level analysis of 20,000 Ontario men followed from the date of an initial negative prostate biopsy demonstrated that PPI use was associated with a 40% increase in prostate cancer mortality rate (16). However, important limitations to these studies, specifically the lack of longitudinal follow-up (15) and the inclusion of a highly select cohort of patients that had undergone a prior work-up for prostate cancer (16), may limit the generalizability of these results, warranting further evaluation of the association of PPI use and prostate cancer outcomes. Accordingly, the objective of this population-based cohort study was to longitudinally estimate the association of cumulative PPI use with prostate cancer diagnosis and adverse outcomes among men with no prior prostate cancer diagnosis, diagnostic work up, or known prior PPI use.

## Materials and Methods

### Study design, setting, and participants

We conducted a population-based cohort study using provincewide-linked administrative health data from Ontario, Canada. These data are routinely and prospectively collected for all health coverage–eligible residents within a publicly funded system that provides universal hospital and physician services, with outpatient prescription drug coverage for individuals ≥65 years. Population-level datasets, including demographics, healthcare utilization, and prescription records, are maintained and linkable at the Institute for Clinical Evaluative Sciences (ICES), an independent, nonprofit research corporation funded by the Ministry of Health and Long-Term Care (17). Ethics approval was obtained from the institutional review boards of Sunnybrook Health Sciences Centre and the University Health Network, Toronto, Canada (18-5770). Written informed consent was not required given the nature of the study. The study was conducted in accordance with the ethical principles of the Declaration of Helsinki.

We identified all men residing in Ontario ages ≥66 years between January 1st, 2003, and December 31st, 2018. Eligible patients had no prior history of a prostate cancer diagnosis, prostate cancer diagnostic work-up [i.e., prostate biopsy and/or transrectal ultrasound (TRUS)], or prostate cancer treatment [i.e., radical prostatectomy, external beam radiotherapy/brachytherapy, androgen deprivation therapy (ADT), and/or bilateral orchiectomy]. Given that Ontario Drug Benefit (ODB) coverage starts at 65 years of age, we applied an age inclusion criteria of ≥66 years to ensure that patients had no prescriptions for PPIs or histamine-2 (H2)-blockers within the year preceding study inclusion. Patients may have received prescriptions prior to age 65 years (ineligible for ODB coverage). All patients were Ontario Health Insurance Plan (OHIP)-eligible for ≥10 years and continuously eligible for ≥1 year prior to study inclusion. Patients were followed until death, administrative censoring, or March 31st, 2020 (the last available follow-up date).

### Study variables and data sources

The primary study exposure was cumulative use of PPIs (esomeprazole, lansoprazole, omeprazole, pantoprazole, and rabeprazole). Given potential confounding secondary to the requirement for anti-reflux medications, H2-blocker use was evaluated as a “positive” control (cimetidine, famotidine, ranitidine, and nizatidine). Exposure to glaucoma eye drops was included as a negative control (brimonidine/timolol and pilocarpine; Supplementary Table S1).

Drug exposure was abstracted from the ODB database, which contains prescription medication claims for those covered under the provincial drug program, including all those ≥65 years of age. Cumulative drug exposure was calculated in 365-day intervals using prescription data, as the product of the number of supply days, frequency of daily use, and a standardization correction factor (PPI dosage standardized to pantoprazole 40 mg once daily-equivalents and H2-blocker dosage standardized to cimetidine 800 mg once-daily equivalents; Supplementary Table S2). Concurrent PPI and H2-blocker use was defined as having concurrent prescriptions for both drugs for ≥90 days (grace period: 30 days). Annual PPI use trends were evaluated over the study period.

The OHIP database was used to capture physician services [i.e., general practitioner (GP) visits, prostate biopsy, TRUS, radiotherapy/brachytherapy (using pelvis-targeted billing codes), and ADT administration]. The Discharge Abstract Database (DAD) was used to identify receipt of a radical prostatectomy, radiotherapy/brachytherapy, and/or bilateral orchiectomy (Supplementary Table S3).

PSA levels were available from the Ontario Laboratories Information System from January 1st, 2012, onward. Biopsy Gleason score (GS) results were available for those with a prostate cancer diagnosis; other pathologic outcomes (e.g., number/percentage of positive cores) were not available. Baseline age and income quintile/rurality were derived from the Registered Persons Database. The cumulative comorbidity burden, quantified via the Johns Hopkins Aggregated Diagnosis Groups (ADG) score from the Johns Hopkins ACG System version 10.0 and specific diagnoses of asthma, chronic obstructive pulmonary disease (COPD), congestive heart failure (CHF), and diabetes were available for each patient. Vital status was ascertained using records from the Office of the Registrar General – Deaths. Cause-specific mortality (e.g., prostate cancer-specific mortality) was not available at the time of analysis.

### Study outcomes

The primary outcome was time to any prostate cancer diagnosis, defined as having either an Ontario Cancer Registry (93% accuracy; refs. 18, 19), DAD, Same Day Surgery, OHIP, or National Ambulatory Care Reporting System record or treatment for prostate cancer, if no diagnosis record was present.

Secondary outcomes were time to (i) clinically significant prostate cancer (definition: GS ≥7), (ii) high-grade prostate cancer (definition: GS ≥8), (iii) first PSA value ≥4 ng/mL among patients who had ≥1 PSA test during follow-up, (iv) first PSA velocity >0.75 ng/mL/year among patients who had ≥2 PSA tests during follow-up, (v) first ADT prescription or bilateral orchiectomy, (vi) first PSA doubling time (PSADT) ≤6 months among patients who had ≥2 PSA tests during follow-up, and (vii) any-cause death. Prostate biopsy procedures were evaluated as a repeated measures outcome in 365-day increments.

### Statistical analysis

Descriptive statistics were summarized for baseline patient characteristics. The associations between the exposures of interest (cumulative PPI and H2-blocker use) and the time-to-event outcomes were evaluated using univariable and multivariable logistic regression analysis with complementary log–log modeling using counting process data in 365-day intervals, which simulates Cox regression modeling, generating hazard ratio (HR) estimates (20). The associations between the study exposures and the odds of a prostate biopsy were evaluated using logistic regression modeling.

All demographic and comorbidity variables were included *a priori* in the multivariable models. The cumulative PPI and H2-blocker variables, along with patient comorbidity variables asthma, COPD, CHF, and diabetes, were modeled as time-varying covariates in 365-day intervals. Cumulative PPI and H2-blocker usage were each operationalized as categorical variables, with nonusers as the referent group and drug users categorized into cumulative dosage quintiles. Baseline income and ADG score were also operationalized as categorical variables (quintiles). Variable collinearity was evaluated using the variance inflation factor test, with a cutoff value of five for collinearity.

Given that more frequent GP visits with subsequent PSA testing may be associated with increased odds of prostate cancer diagnosis, we performed sensitivity analyses for the associations between cumulative PPI and H2-blocker use and prostate cancer diagnosis, adjusting for the frequency of GP visits and PSA tests performed in the prior 2 years. These variables were operationalized as time-varying covariates. These sensitivity analyses were restricted to patients with PSA tests performed (January 1st, 2012, onward).

The annual PPI and H2-blocker use trends were calculated by dividing the total number of users for each drug by the total number of patients, per year.

No power calculations were performed, with all men meeting the study eligibility criteria included. Statistical significance was set at *P* < 0.05. All statistical analyses were performed using SAS Enterprise Guide version 8.3 (SAS institute).

## Results

### Cohort characteristics

The study cohort included 559,425 men (Supplementary Fig. S1). The median follow-up duration was 9.2 years [interquartile range (IQR): 5–14.5]. The median age was 66 years (IQR: 66–69). Any PPI and H2-blocker use during follow-up was recorded in 168,890 (30.2%) and 42,939 (7.7%) men, respectively (Table 1). Compared with nonusers, PPI and/or H2-blocker users had a greater comorbidity burden (ADG score ≥7: 16.8% vs. 28%–34%) and more frequent GP visits in the 2 years prior to the index date (median: 4 vs. 8–9 visits).

**Table 1.** Baseline characteristics.

​	Overall^a^ (*n* = 559,425)	Both PPIs and H2-blockers (*n* = 27,571)	PPIs only (*n* = 141,319)	H2-blockers only (*n* = 15,368)	Non–drug use (*n* = 354,962)
Variable	​	​	​	​	​
Age, median (IQR)	66 (66–69)	66 (66–70)	66 (66–69)	66 (66–71)	66 (66–69)
Income quintile/rurality, *n* (%)	​	​	​	​	​
1 (lowest)	80,366 (14.4%)	3,783 (13.7%)	18,189 (12.9%)	2,128 (13.8%)	53,701 (15.1%)
2	91,981 (16.4%)	4,551 (16.5%)	22,901 (16.2%)	2,468 (16.1%)	58,886 (16.6%)
3	92,476 (16.5%)	4,685 (17%)	23,855 (16.9%)	2,576 (16.8%)	58,123 (16.4%)
4	94,165 (16.8%)	4,578 (16.6%)	24,427 (17.3%)	2,568 (16.7%)	59,040 (16.6%)
5 (highest)	105,237 (18.8%)	4,695 (17%)	26,387 (18.7%)	2,695 (17.5%)	66,959 (18.9%)
Rural	93,083 (16.6%)	5,160 (18.7%)	25,080 (17.7%)	2,877 (18.7%)	56,864 (16%)
Missing	2,117 (0.4%)	119 (0.4%)	480 (0.3%)	56 (0.4%)	1,389 (0.4%)
ADG (*n*, %)	​	​	​	​	​
0	105,946 (18.9%)	1,415 (5.1%)	9,253 (6.5%)	1,116 (7.3%)	92,544 (26.1%)
1–2	103,602 (18.5%)	3,849 (14%)	23,595 (16.7%)	2,698 (17.6%)	69,360 (19.5%)
3–4	126,515 (22.6%)	6,322 (22.9%)	34,562 (24.5%)	3,786 (24.6%)	76,488 (21.5%)
5–6	103,774 (18.6%)	6,598 (23.9%)	32,393 (22.9%)	3,461 (22.5%)	56,941 (16%)
7 or higher	119,588 (21.4%)	9,387 (34%)	41,516 (29.4%)	4,307 (28%)	59,629 (16.8%)
Baseline asthma (*n*, %)	32,911 (5.9%)	2,270 (8.2%)	10,409 (7.4%)	1,041 (6.8%)	18,073 (5.1%)
Baseline COPD (*n*, %)	67,014 (12%)	4,222 (15.3%)	19,947 (14.1%)	2,338 (15.2%)	38,654 (10.9%)
Baseline CHF (*n*, %)	20,333 (3.6%)	920 (3.3%)	5,388 (3.8%)	703 (4.6%)	12,814 (3.6%)
Baseline diabetes (*n*, %)	100,781 (18%)	5,608 (20.3%)	29,875 (21.1%)	2,910 (18.9%)	58,276 (16.4%)
Number of GP visits in the 2 years prior to the index date, median (IQR)	6 (1–11)	9 (5–15)	8 (4–14)	8 (3–14)	4 (0–9)
Number of PSA tests in 2 years prior to the index date, mean (SD)	0.2 (0.5)	0.1 (0.4)	0.2 (0.5)	0.1 (0.4)	0.2 (0.5)

Abbreviation: SD, standard deviation.

a Glaucoma eye drop users accounted for the remaining 20,205 cohort subjects (3.6%).

**Figure 1.**
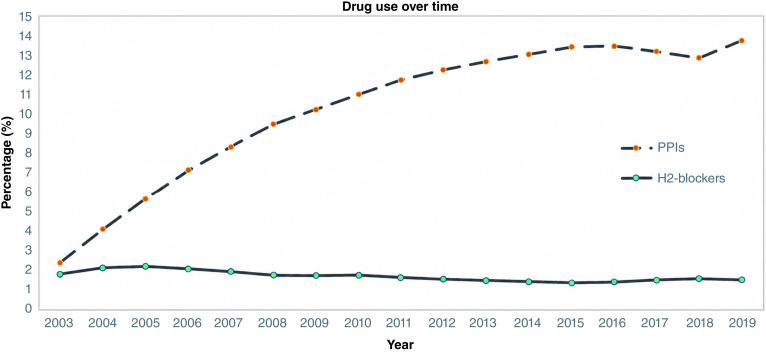
Annual PPI and H2-blocker drug use over the study period.

The annual percentage of PPI users steadily increased from 2.4% in 2003 to 13.8% in 2019, whereas it remained stable for H2-blocker users (1.8% in 2003 vs. 1.6% in 2019; Fig. 1; Supplementary Table S4).

### Prostate cancer diagnosis

Overall, 46,799 men (8.4%) were diagnosed with prostate cancer. On multivariable analysis adjusted for age, income quintile, and patient comorbidities (ADG score and individual comorbidities), cumulative PPI use was associated with progressively lower rates of a prostate cancer diagnosis [HRs for lowest and highest PPI user quintiles, compared with non–drug users: 0.91 (95% confidence interval (CI): 0.88–0.95) and 0.75 (0.71–0.79), respectively; *P* < 0.001 for both].

Sensitivity analyses further adjusting for the frequency of GP visits and PSA tests performed demonstrated that cumulative PPI use was no longer significantly associated with the rates of prostate cancer diagnosis (HR for highest user quintile vs. nonusers: 0.99, 95% CI, 0.93–1.05; *P* = 0.68). Each additional 10 GP visits and one PSA test performed within the preceding 2 years was associated with 7% and 90% increased rates of a prostate cancer diagnosis [HRs, 1.07 (1.06–1.08) and 1.90 (1.88–1.91), respectively; *P* < 0.001 for both].

Patients in the highest H2-blocker use quintile had significantly lower rates of a prostate cancer diagnosis, compared with nonusers (HR, 0.85; 95% CI, 0.77–0.93; *P* < 0.001), but this association was similarly no longer significant on the sensitivity analysis (HR, 0.95; 95% CI, 0.84–1.07; *P* = 0.39; Table 2; Supplementary Table S5).

**Table 2.** Multivariable logistic regression analysis (with complementary log–log link) for the outcome of prostate cancer diagnosis, using counting process data, by time-varying exposure of drug quintile^a^.

Variable	HR	95% CI	*P* value
Model not adjusting for frequency of GP visits and PSA tests within prior 2 years			
PPI use quintile (Referent: non–drug users)	​	​	​
1st (lowest)	0.91	0.88–0.95	<0.001
2nd	0.89	0.85–0.94	<0.001
3rd	0.85	0.81–0.90	<0.001
4th	0.83	0.79–0.88	<0.001
5th (highest)	0.75	0.71–0.79	<0.001
H2-blocker use quintile (Referent: non–drug users)	​	​	​
1st (lowest)	0.93	0.84–1.02	0.13
2nd	1.01	0.94–1.10	0.75
3rd	0.96	0.88–1.05	0.35
4th	0.95	0.87–1.04	0.27
5th (highest)	0.85	0.77–0.93	<0.001
Model adjusting for frequency of GP visits and PSA tests within prior 2 years			
PPI use quintile (Referent: non–drug users)	​	​	​
1st (lowest)	1.09	1.03–1.15	0.002
2nd	1.07	1–1.14	0.047
3rd	1.01	0.95–1.08	0.65
4th	1.01	0.95–1.07	0.83
5th (highest)	0.99	0.93–1.05	0.68
H2-blocker use quintile (Referent: non–drug users)	​	​	​
1st (lowest)	0.98	0.86–1.11	0.73
2nd	0.97	0.87–1.08	0.57
3rd	1	0.89–1.12	0.96
4th	0.90	0.80–1.02	0.09
5th (highest)	0.95	0.84–1.07	0.39
GP visits in prior 2 years	1.07	1.06–1.08	<0.001
PSA tests in prior 2 years	1.90	1.88–1.91	<0.001

a Adjusted for income quintile, ADG score, presence/absence of asthma, COPD, CHF, diabetes, and age, which was operationalized as a categorical variable with each stratum representing an age quarter, mimicking Cox model results.

### Clinically significant and high-grade prostate cancer

Clinically significant and high-grade prostate cancer were diagnosed in 8,701 (1.6%) and 3,408 (0.61%) men, respectively. On multivariable analysis, neither cumulative PPI nor H2-blocker use was associated with the rates of clinically significant or high-grade prostate cancer (Table 3; Supplementary Tables S6 and S7).

**Table 3.** Multivariable logistic regression analyses (with complementary log–log link) for the outcomes of clinically significant (i.e., GS ≥7) and high-grade prostate cancer (i.e., GS ≥8), using counting process data, by time-varying exposure of drug quintile^a^.

Variable	HR	95% CI	*P* value
Clinically significant prostate cancer			
PPI use quintile (Referent: non–drug users)	​	​	​
1st (lowest)	1.11	1.01–1.21	0.03
2nd	1.08	0.97–1.21	0.17
3rd	1.02	0.92–1.13	0.74
4th	1.03	0.93–1.14	0.57
5th (highest)	0.99	0.89–1.10	0.84
H2-blocker use quintile (Referent: non–drug users)	​	​	​
1st (lowest)	0.86	0.68–1.07	0.18
2nd	0.87	0.72–1.06	0.16
3rd	0.98	0.80–1.19	0.72
4th	0.82	0.66–1.01	0.07
5th (highest)	0.91	0.74–1.12	0.37
High-grade prostate cancer			
PPI use quintile (Referent: non–drug users)	​	​	​
1st (lowest)	1.05	0.90–1.21	0.55
2nd	1.07	0.90–1.27	0.43
3rd	0.92	0.78–1.09	0.33
4th	0.94	0.79–1.11	0.45
5th (highest)	1.04	0.88–1.21	0.67
H2-blocker use quintile (Referent: non–drug users)	​	​	​
1st (lowest)	0.98	0.70–1.36	0.90
2nd	0.95	0.71–1.26	0.72
3rd	1.08	0.81–1.45	0.59
4th	0.91	0.67–1.26	0.58
5th (highest)	1.10	0.82–1.46	0.53

a Adjusted for income quintile, ADG score, presence/absence of asthma, COPD, CHF, diabetes, and age, which was operationalized as a categorical variable with each stratum representing an age quarter, mimicking Cox model results.

### Time to first PSA ≥4 ng/mL and >0.75 ng/mL/year

A PSA ≥4 ng/mL was observed in 71,621 of 277,991 (25.8%) men who had ≥1 PSA test performed. On multivariable analysis, cumulative PPI use was associated with progressively lower rates of a first serum PSA level ≥4 ng/mL (HR for highest use quintile vs. nonusers: 0.93, 95% CI, 0.93–0.94; *P* < 0.001). The corresponding HRs for H2-blocker users, compared with nonusers, ranged from 0.91 to 1.03 (Supplementary Tables S8 and S9).

A PSA velocity >0.75 ng/mL/year was observed in 62,920 of 197,914 (31.8%) men who had ≥2 PSA tests performed. On multivariable analysis, no consistent association was observed between cumulative PPI use and the rates of a first PSA velocity >0.75 ng/mL/year (HRs, 0.92–1.02; Supplementary Tables S10 and S11).

### Prostate biopsy

During the study period, 33,183 (5.5%) men underwent ≥1 prostate biopsy (Supplementary Table S12). On multivariable analysis, cumulative PPI use was associated with increased odds of a prostate biopsy for the lowest quintile users [odds ratios (OR) for first and second user quintiles: 1.16 (1.11–1.23) and 1.14 (1.07–1.22), respectively, *P* < 0.001 for both] but lower odds for the highest quintile users (HR, 0.85; 95% CI, 0.79–0.92; *P* < 0.001). No consistent association was observed between cumulative H2-blocker use and odds of undergoing a prostate biopsy (Supplementary Tables S13 and S14).

### Time to first ADT prescription or bilateral orchiectomy and PSADT ≤6 months

Overall, 15,827 (2.8%) men received an ADT prescription or underwent a bilateral orchiectomy. On multivariable analysis, cumulative PPI use was associated with progressively lower rates of a first ADT prescription or bilateral orchiectomy (HR for highest PPI user quintile: 0.83, 95% CI, 0.77–0.90; *P* < 0.001). No significant association was observed between cumulative H2-blocker use and time to first ADT prescription or bilateral orchiectomy (Table 4; Supplementary Table S15).

**Table 4.** Multivariable logistic regression analysis (with complementary log–log link) for the outcome of the first ADT prescription or bilateral orchiectomy, using counting process data, by time-varying exposure of drug quintile^a^.

Variable	HR	95% CI	*P* value
PPI use quintile (Referent: non–drug users)	​	​	​
1st (lowest)	1	0.94–1.07	0.97
2nd	0.92	0.85–1	0.054
3rd	0.88	0.81–0.95	0.001
4th	0.87	0.80–0.94	<0.001
5th (highest)	0.83	0.77–0.90	<0.001
H2-blocker use quintile (Referent: non–drug users)	​	​	​
1st (lowest)	0.92	0.79–1.08	0.29
2nd	1	0.88–1.14	0.99
3rd	0.94	0.82–1.09	0.40
4th	0.95	0.83–1.10	0.51
5th (highest)	0.88	0.76–1.02	0.083

a Adjusted for income quintile, ADG score, presence/absence of asthma, COPD, CHF, diabetes, and age, which was operationalized as a categorical variable with each stratum representing an age quarter, mimicking Cox model results.

Any PPI use was associated with a 3% to 12% increased rate of a first PSADT ≤6 months on univariable analysis; however, cumulative PPI use was not associated with progressively increasing event rates on multivariable analysis (HRs, 1.02–1.11; *P* for all < 0.001). Similarly, no consistent association was observed between H2-blocker use and the rate of a first PSADT ≤6 months (Supplementary Tables S16 and S17).

### Any-cause death

Overall, 155,487 (27.8%) men died during the study follow-up. Glaucoma eye drops use, the negative control, was not associated with any-cause death (Supplementary Tables S18 and S19). Compared with never-users, PPI and H2-blocker use trends were both significantly associated with increased any-cause death rates (HRs, 1.11–1.36 and 1.10–1.20, respectively; Supplementary Tables S18–S23).

## Discussion

In this population-based cohort study of 559,425 men ages ≥66 years with no previous diagnosis or diagnostic work-up for prostate cancer and no prior exposure to PPIs or H2-blockers, there was no association observed between cumulative PPI use and the rates of prostate cancer diagnosis, after adjustment for patient comorbidities, income quintile, and the frequency of GP visits and PSA tests performed within the preceding 2 years in men with available PSA levels. Similarly, cumulative PPI use was not associated with the rates of clinically significant or high-grade prostate cancer.

Our results highlight the importance of considering all possible confounders, as well as potential mediators and colliders, of an association between a drug exposure and the outcome(s) of interest. Our initial multivariable model demonstrated that cumulative PPI use was associated with up to 25% lower rates of a prostate cancer diagnosis. However, it has been well-documented that the incidence of prostate cancer diagnosis is closely correlated with the frequency of PSA testing (21, 22), and patients prescribed PPIs or H2-blockers by healthcare providers may have more frequent encounters with the healthcare system that may result in disparate frequencies of PSA testing and, thus, prostate cancer diagnosis. Significantly, following adjustment for these surrogates of healthcare resource utilization, the previously demonstrated protective effect of PPIs was “neutralized”, and there was no significant association observed between PPI use and the rates of prostate cancer diagnosis (Table 2). PSA testing and healthcare utilization may lie on the causal pathway between PPI use and prostate cancer detection, functioning as mediators or potential colliders.

Similar findings observed for H2-blocker use, a positive control exposure with overlapping clinical indications and healthcare utilization patterns, further support the interpretation that the initially observed associations were likely driven by differences in healthcare engagement and cancer detection. Additionally, glaucoma eye drop use, included as a negative control exposure, was not associated with any-cause mortality, providing reassurance against substantial systematic bias.

We did note, however, that cumulative PPI use was associated with up to 17% lower rates of a first ADT prescription or bilateral orchiectomy, classically used surrogates of advanced disease (although may be administered concomitantly with radiotherapy for localized disease). PPIs block H+ transporters, including vacuolar ATPase (V-ATPase) proton pumps, which acidify the extracellular space and increase tumor-cell motility, proliferation, multidrug resistance, and metastasis. V-ATPase inhibition has been shown to reduce the *in vitro* invasion of LNCaP and PC-3 cells by 80% and PSA mRNA expression in the androgen-dependent LNCaP cells (23). Additionally, emerging evidence is linking gut microbiome alterations to prostate cancer pathogenesis (24, 25), and PPIs are known to alter the gut microbiome (26, 27), which may provide an additional explanation for the observed association. Nonetheless, these findings remain only hypothesis-generating in nature, and further investigations of potential mechanisms underlying such an association are needed.

Limitations to this study include the lack of cause of death (i.e., prostate cancer-specific mortality). Available pathologic data were limited to GS and did not include other pathologic features (e.g., volume of disease, presence of cribriform/intraductal patterns), which may have resulted in incomplete risk stratification. Serum PSA levels were only available from 2012 onward, limiting adjustment for detection-related factors across the full cohort. Detailed staging information and mode of diagnosis (e.g., biopsy, TURP/HoLEP, or clinical/imaging-based diagnosis) were not available within the administrative datasets. As such, we were unable to distinguish diagnostic pathways or identify incidentally detected, low-risk disease (e.g., pT1a), which may have influenced observed incidence rates.

Although a 1-year look-back window was applied, prior intermittent PPI or H2-blocker use before age 65 years may have occurred, resulting in potential nondifferential exposure misclassification and bias toward the null. The association between PPIs and adverse prostate cancer outcomes was not stratified by specific PPI drug, which may mask drug-specific effects.

The exact indication for drug use and underlying disease (e.g., GERD) severity were not available. Potential residual confounders, including GERD severity, obesity, smoking status, and diet, were not adjusted for in our analyses. No methods were applied to reduce the risk of reverse causation.

This analysis was restricted to men ≥66 years of age, which limits the generalizability of these results to younger patient cohorts. The median study follow-up of 9.2 years may be insufficient to detect long-latency effects. Additionally, exclusion of men with any prior prostate cancer investigation may have enriched the cohort for individuals at lower baseline risk, potentially underestimating true incidence rates and limiting generalizability to populations undergoing routine diagnostic evaluation.

A complementary log–log logistic regression model, as opposed to a Cox proportional hazard model, was used to for this analysis due to practical limitations with the SAS software processing abilities, given the large sample size >500,000 men. Output from such models has been shown to reliably mimic those obtained from Cox models, with similar interpretations (19). Finally, this analysis was generated using retrospective data from health administrative database, which may be subject to information biases/misclassification errors. However, prospective, randomized evaluation of PPI-associated carcinogenic effects is pragmatically not feasible.

## Supplementary Material

Supplementary Table 1 Drug identification number list

Supplementary Table 2 Cumulative drug exposure calculations

Supplementary Table 3 Codes for OHIP and CIHI DAD abstracted data

Supplementary Table 4 Percentage of drug use over year, based on unique patient data

Supplementary Table 5 Univariable logistic regression analysis (with complementary log-log link) for the outcome of prostate cancer diagnosis, using counting process data, by time-varying exposure of drug quintile

Supplementary Table 6 Univariable logistic regression analysis (with complementary log-log link) for the outcome of clinically significant prostate cancer diagnosis (i.e., Gleason Score ≥7), using counting process data, by time-varying exposure of drug quintile

Supplementary Table 7 Univariable logistic regression analysis (with complementary log-log link) for the outcome of high-grade prostate cancer diagnosis (i.e., Gleason Score ≥8), using counting process data, by time-varying exposure of drug quintile

Supplementary Table 8 Univariable logistic regression analysis (with complementary log-log link) for the outcome of first PSA value ≥4 ng/ml, using counting process data, by time-varying exposure of drug quintile, among patients with ≥1 PSA test after index date

Supplementary Table 9 Multivariable logistic regression analysis (with complementary log-log link) for the outcome of first PSA value ≥4 ng/ml, using counting process data, by time-varying exposure of drug quintile, among patients with ≥1 PSA test after index date

Supplementary Table 10 Univariable logistic regression analysis (with complementary log-log link) for the outcome of first PSA velocity >0.75 ng/ml/year, using counting process data, by time-varying exposure of drug quintile, among patients with ≥2 PSA test after index date

Supplementary Table 11 Multivariable logistic regression analysis (with complementary log-log link) for the outcome of first PSA velocity >0.75 ng/ml/year, using counting process data, by time-varying exposure of drug quintile, among patients with ≥2 PSA test after index date

Supplementary Table 12 Distribution of biopsy by drug use, based on unique patient data

Supplementary Table 13 Univariable logistic regression analysis for the outcome of prostate biopsy, using counting process data, by time-varying exposure of drug quintile

Supplementary Table 14 Multivariable logistic regression analysis for the outcome of prostate biopsy, using counting process data, by time-varying exposure of drug quintile

Supplementary Table 15 Univariable logistic regression analysis (with complementary loglog link) for the outcome of the first ADT prescription or bilateral orchiectomy, using counting process data, by time-varying exposure of drug quintile

Supplementary Table 16 Univariable logistic regression analysis (with complementary loglog link) for the outcome of the first PSA doubling time ≤6 months, using counting process data, by time-varying exposure of drug quintile

Supplementary Table 17 Multivariable logistic regression analysis (with complementary loglog link) for the outcome of the first PSA doubling time ≤6 months, using counting process data, by time-varying exposure of drug quintile

Supplementary Table 18 Univariable logistic regression analysis (with complementary loglog link) for the outcome of the any-cause death, using counting process data, by time-varying exposure of drug quintile

Supplementary Table 19 Multivariable logistic regression analysis (with complementary loglog link) for the outcome of the any-cause death, using counting process data, by time-varying exposure of drug quintile

Supplementary Table 20 Summary of patients, outcome events and records

Supplementary Table 21 Person-time rates of study outcomes, by exposure, based on the counting process data

Supplementary Table 22 Drug dosage by quintile for the study outcomes, based on the counting process data

Supplementary Table 23 STROBE Checklist

Supplementary Figure 1 Study flow chart

## Data Availability

The data sets generated and/or analyzed during the current study are not publicly available due to Ontario provincial regulations governing the privacy and protection of patient-level health administrative data. Access to these data may be granted to qualified individuals through the ICES, subject to data sharing agreements, institutional approvals, and applicable privacy regulations.
