# Supplementary material for: Association of Cumulative Proton Pump Inhibitor Use with Prostate Cancer Risk and Outcomes: A Population-Based Cohort Study
Source: Cancer Res Commun. 2026 Jul 24;6(7):1769–76. doi: 10.1158/2767-9764.CRC-26-0098 (PMC13396002; doi:10.1158/2767-9764.CRC-26-0098)
Supplement: Supplementary Table 1 — Drug identification number list [file crc-26-0098_supplementary_table_1_suppst1.docx]

| **Supplementary Table 1. Drug identification number list** | |
| --- | --- |
| Androgen deprivation therapy – LHRH agonists | Buserelin acetate (Suprefact)  02225166 (Suprefact 1mg), 02225158 (Suprafact 1mg),  02228955 (Suprefact depot 2mo 6.3mg), 02240749 (Suprefact depot 3mo 9.45mg)  Leuprolide acetate (Lupron, Eligard)  00727695(Lupron 5mg),00884502 (Lupron depot 3.75mg), 00836273 (Lupron depot 7.5mg),  02239834 (Lupron depot 11.25mg),02230248 (Lupron depot 22.5mg), 02239833 (Lupron depot 30mg),  02248239 (Eligard 7.5mg),02248240 (Eligard 22.5mg)  02248999 (Eligard 30mg),02268892 (Eligard 45mg)  Goserelin acetate (Zoladex)  02049325 (Zoladex 3.6mg), 02225905 (Zoladex 10.8mg)  Triptorelin pamoate (Trelstar)  02240000 (Trelstar 3.75mg),02243856 (Trelstar 11.25mg),02412322 (Trelstar 22.5mg)  Histrelin acetate (Vantas)  02278383 |
| Androgen deprivation therapy – LHRH antagonists | Degarelix acetate (Firmagon)  02337029 (Firmagon 80mg),02337037 (Firmagon 120mg) |
| Androgen deprivation therapy - antiandrogens | 02238560, 0637726, 02296063, 02325985, 02184478, 02274337, 02357216, 02270226, 02275589, 02371324, 02276089, 02428709, 02221861, 0704431, 02245898, 02390760, 02290308, 02233542, 02309556, 02425017 |
| Proton pump inhibitors - esomepraozle | 02423863, 02339102, 02383047, 02244522, 02379171  02423987, 02423855, 02339099, 02383039, 02244521  02423979, 02458608, 02443449, 02361701, 02361728 |
| Proton pump inhibitors - rabeprazole | 02345579, 02345587, 02408392, 02408406, 02310805  02310813, 02243797, 02243796, 02298082, 02298074  02314185, 02314177, 02296640, 02296632 |
| Proton pump inhibitors - pantoprazole | 02408570, 02441853, 02267233, 02440628, 02292912  02292920, 02415208, 02300486, 02408414, 02357054  02416565, 02416557, 02417448, 02299585, 02285487  02285479, 02307871, 02241804, 02229453, 02437945  02305038, 02305046, 02301083, 02301075, 02428164 |
| Proton pump inhibitors - lansoprazole | 02293838, 02293811, 02433001, 02433028, 02353830, 02353849, 02280515, 02280523, 02165503, 02165511,02402610, 02402629, 02385643, 02385651, 02238525 |
| Proton pump inhibitors - omeprazole | 02423863, 02339102, 02383047, 02244522, 02379171  02423987, 02423855, 02339099, 02383039, 02244521  02423979, 02443449, 02458608, 02361728, 02361701  02245058, 09857285, 02420198, 0846503, 02190915  02230737, 02329433, 09857341, 09857350, 02439549  02295415, 02416549, 09857342, 02320851, 02310260  02403617, 02374870, 02260867, 02296446, 09857314  02295407, 02432404, 02119579, 02329425, 02296438  09857464, 09857195, 09857343, 09857536, 09857500  09857267, 09857530 |
| Glaucoma eye drops - brimonidine/timolol | 02236876, 02248151, 02260077, 02301334, 02246284  02305429, 09857298, 02435411, 09857513, 09857298  02451271, 02443090, 02442426, 02437686, 02436256  02414155, 02404389, 02394685, 02373068, 02344351  02331624, 02299615, 02290812, 02258692, 02246619  02242276, 02242275, 02240113, 02171899, 02171880  02166720, 02166712, 02083353, 02083345, 00755834  00755826, 00451207,00451193 |
| Glaucoma eye drops - pilocarpine | 02023741, 02023725, 00000868, 00000884, 00000841 |
| H2 blockers Famotidine | 02509970, 02509989, 02231119, 02244888, 02244998, 01953842, 01953834, 02257645, 02257653, 02238212, 02244133, 02246364, 02247745, 02247735, 02247735, 02507749, 02507757, 02022133, 02022141 |
| H2 blockers Cimetidine | 02243085, 00487872, 00584215, 00600059, 00600067, 00749494 |
| H2 blockers Ranitidine | 02248570, 02248571, 02484501, 02484528, 02280833, 00733059, 00733067, 02243038, 02243039, 02463717, 02463725, 02473542, 02443708, 02443716, 02242453, 02242454, 02494310, 02494329, 02336480, 02336502, 00740748, 00740756, 02245615, 02353016, 02353024, 02385953, 02385961, 02350203, 02350211 |
| H2 blockers Nizatidine | 02185814, 02520338, 02520346 |

H2: Histamine-2

LHRH: Luteinizing hormone releasing hormone
