# Supplementary material for: Association of Cumulative Proton Pump Inhibitor Use with Prostate Cancer Risk and Outcomes: A Population-Based Cohort Study
Source: Cancer Res Commun. 2026 Jul 24;6(7):1769–76. doi: 10.1158/2767-9764.CRC-26-0098 (PMC13396002; doi:10.1158/2767-9764.CRC-26-0098)
Supplement: Supplementary Table 2 — Cumulative drug exposure calculations [file crc-26-0098_supplementary_table_2_suppst2.docx]

| **Supplementary Table 2.** Cumulative drug exposure calculations | | |
| --- | --- | --- |
| Class of Drugs | Drug | Correction Factor |
| PPI* | Pantoprazole | Reference |
|  | Esomeprazole | 2 |
|  | Lansoprazole | 1.33 |
|  | Omeprazole | 2 |
|  | Rabeprazole | 2 |
| H2-blocker** | Famotidine | Reference |
|  | Cimetidine | 0.05 |
|  | Ranitidine | 0.1333 |
|  | Nizatidine | 0.1333 |

*Pantoprazole equivalents

**Famotidine equivalents
