# Supplementary material for: Association of Cumulative Proton Pump Inhibitor Use with Prostate Cancer Risk and Outcomes: A Population-Based Cohort Study
Source: Cancer Res Commun. 2026 Jul 24;6(7):1769–76. doi: 10.1158/2767-9764.CRC-26-0098 (PMC13396002; doi:10.1158/2767-9764.CRC-26-0098)
Supplement: Supplementary Table 3 — Codes for OHIP and CIHI DAD abstracted data [file crc-26-0098_supplementary_table_3_suppst3.docx]

| **Supplementary Table 3.** Codes for OHIP and CIHI DAD abstracted data | |
| --- | --- |
| **Prostate Cancer** | ICD-9: 185, ICD-10: C61  ICD-O: C61.9 |
|  |  |
| **OHIP** | **Code** |
| Prostate Biopsy | Z712, Z713, S644, E780 |
| Transrectal ultrasound | J128, J135, J138, J149, J162, J180 |
| Radiation therapy for PCa (pelvis-specific) | X336, X310, X311, X312, X313, X322 |
| Brachytherapy | S640 till 2007, afterwards X323, X324, X325 |
| Androgen Deprivation Therapy | G342 |
| **CIHI DAD** | **Code** |
| Bilateral orchiectomy | CCP Code: 74.31; CCI Code: 1QM89 |
| Radical prostatectomy | CCP: 72.4, CCI: 1QT91 |
| Radiation/brachytherapy | CCP: 0634, CCI: 1QT26 or 1QT53 |

CIHI DAD: Canadian Institute for Health Information Discharge Abstract Database

OHIP: Ontario Health Insurance Plan
