# Supplementary material for: Association of Cumulative Proton Pump Inhibitor Use with Prostate Cancer Risk and Outcomes: A Population-Based Cohort Study
Source: Cancer Res Commun. 2026 Jul 24;6(7):1769–76. doi: 10.1158/2767-9764.CRC-26-0098 (PMC13396002; doi:10.1158/2767-9764.CRC-26-0098)
Supplement: Supplementary Table 4 — Percentage of drug use over year, based on unique patient data [file crc-26-0098_supplementary_table_4_suppst4.docx]

| **Supplementary Table 4. Percentage of drug use over year, based on unique patient data** | | | | | |
| --- | --- | --- | --- | --- | --- |
| **Year** | **Number of PPI users** | **Number of H2-blocker users** | **Total number of patients** | **% of PPI users** | **% H2-blocker users** |
| 2003 | 4,938 | 3,742 | 204,206 | 2.42 | 1.83 |
| 2004 | 9,132 | 4,784 | 221,828 | 4.12 | 2.16 |
| 2005 | 13,499 | 5,285 | 237,746 | 5.68 | 2.22 |
| 2006 | 18,093 | 5,317 | 252,825 | 7.16 | 2.10 |
| 2007 | 22,225 | 5,248 | 266,856 | 8.33 | 1.97 |
| 2008 | 26,604 | 5,018 | 280,807 | 9.47 | 1.79 |
| 2009 | 30,143 | 5,208 | 295,017 | 10.22 | 1.77 |
| 2010 | 33,929 | 5,495 | 308,770 | 10.99 | 1.78 |
| 2011 | 37,773 | 5,320 | 321,892 | 11.73 | 1.65 |
| 2012 | 41,479 | 5,353 | 338,831 | 12.24 | 1.58 |
| 2013 | 45,235 | 5,379 | 356,909 | 12.67 | 1.51 |
| 2014 | 48,664 | 5,411 | 373,378 | 13.03 | 1.45 |
| 2015 | 52,191 | 5,417 | 389,110 | 13.41 | 1.39 |
| 2016 | 54,408 | 5,821 | 404,135 | 13.46 | 1.44 |
| 2017 | 55,149 | 6,467 | 418,326 | 13.18 | 1.55 |
| 2018 | 55,659 | 6,992 | 432,863 | 12.86 | 1.62 |
| 2019 | 57,788 | 6,537 | 420,278 | 13.75 | 1.56 |

H2: Histamine-2

PPI: Proton pump inhibitor
