# Supplementary material for: Association of Cumulative Proton Pump Inhibitor Use with Prostate Cancer Risk and Outcomes: A Population-Based Cohort Study
Source: Cancer Res Commun. 2026 Jul 24;6(7):1769–76. doi: 10.1158/2767-9764.CRC-26-0098 (PMC13396002; doi:10.1158/2767-9764.CRC-26-0098)
Supplement: Supplementary Table 6 — Univariable logistic regression analysis (with complementary log-log link) for the outcome of clinically significant prostate cancer diagnosis (i.e., Gleason Score ≥7), using counting process data, by time-varying exposure of drug quintile [file crc-26-0098_supplementary_table_6_suppst6.docx]

| **Supplementary Table 6. Univariable logistic regression analysis (with complementary log-log link) for the outcome of clinically significant prostate cancer diagnosis (i.e., Gleason Score ≥7), using counting process data, by time-varying exposure of drug quintile^a^** | | | |
| --- | --- | --- | --- |
| **Variable** | **Hazard Ratio** | **95% Confidence Interval** | **P-Value** |
| PPI use quintile  (Referent: Non-drug users) |  |  |  |
| 1^st^ (Lowest) | 1.21 | 1.11–1.33 | <0.001 |
| 2^nd^ | 1.17 | 1.05–1.31 | 0.005 |
| 3^rd^ | 1.09 | 0.98–1.21 | 0.10 |
| 4^th^ | 1.09 | 0.98–1.21 | 0.10 |
| 5^th^ (Highest) | 1.05 | 0.94–1.17 | 0.40 |
| H2-blocker use quintile  (Referent: Non-drug users) |  |  |  |
| 1^st^ (Lowest) | 0.94 | 0.75–1.18 | 0.58 |
| 2^nd^ | 0.95 | 0.79–1.15 | 0.61 |
| 3^rd^ | 1.07 | 0.88–1.30 | 0.50 |
| 4^th^ | 0.88 | 0.71–1.09 | 0.25 |
| 5^th^ (Highest) | 0.97 | 0.79–1.20 | 0.80 |

^a^Adjusted for age, operationalized as a categorical variable with each stratum representing an age quarter, mimicking Cox model results

H2: Histamine-2

PPI: Proton pump inhibitor
