# Supplementary material for: Association of Cumulative Proton Pump Inhibitor Use with Prostate Cancer Risk and Outcomes: A Population-Based Cohort Study
Source: Cancer Res Commun. 2026 Jul 24;6(7):1769–76. doi: 10.1158/2767-9764.CRC-26-0098 (PMC13396002; doi:10.1158/2767-9764.CRC-26-0098)
Supplement: Supplementary Table 7 — Univariable logistic regression analysis (with complementary log-log link) for the outcome of high-grade prostate cancer diagnosis (i.e., Gleason Score ≥8), using counting process data, by time-varying exposure of drug quintile [file crc-26-0098_supplementary_table_7_suppst7.docx]

| **Supplementary Table 7. Univariable logistic regression analysis (with complementary log-log link) for the outcome of high-grade prostate cancer diagnosis (i.e., Gleason Score ≥8), using counting process data, by time-varying exposure of drug quintile^a^** | | | |
| --- | --- | --- | --- |
| **Variable** | **Hazard Ratio** | **95% Confidence Interval** | **P-Value** |
| PPI use quintile  (Referent: Non-drug users) |  |  |  |
| 1^st^ (Lowest) | 1.14 | 0.99–1.32 | 0.07 |
| 2^nd^ | 1.16 | 0.98–1.38 | 0.08 |
| 3^rd^ | 0.99 | 0.84–1.17 | 0.92 |
| 4^th^ | 1.00 | 0.85–1.18 | 0.98 |
| 5^th^ (Highest) | 1.11 | 0.96–1.30 | 0.17 |
| H2-blocker use quintile  (Referent: Non-drug users) |  |  |  |
| 1^st^ (Lowest) | 1.05 | 0.76–1.46 | 0.76 |
| 2^nd^ | 1.01 | 0.76–1.35 | 0.92 |
| 3^rd^ | 1.16 | 0.87–1.55 | 0.32 |
| 4^th^ | 0.97 | 0.70–1.33 | 0.83 |
| 5^th^ (Highest) | 1.16 | 0.88–1.54 | 0.30 |

^a^Adjusted for age, operationalized as a categorical variable with each stratum representing an age quarter, mimicking Cox model results

H2: Histamine-2

PPI: Proton pump inhibitor
