# Supplementary material for: Association of Cumulative Proton Pump Inhibitor Use with Prostate Cancer Risk and Outcomes: A Population-Based Cohort Study
Source: Cancer Res Commun. 2026 Jul 24;6(7):1769–76. doi: 10.1158/2767-9764.CRC-26-0098 (PMC13396002; doi:10.1158/2767-9764.CRC-26-0098)
Supplement: Supplementary Table 8 — Univariable logistic regression analysis (with complementary log-log link) for the outcome of first PSA value ≥4 ng/ml, using counting process data, by time-varying exposure of drug quintile, among patients with ≥1 PSA test after index date [file crc-26-0098_supplementary_table_8_suppst8.docx]

| **Supplementary Table 8. Univariable logistic regression analysis (with complementary log-log link) for the outcome of first PSA value ≥4 ng/ml, using counting process data, by time-varying exposure of drug quintile, among patients with ≥1 PSA test after index date^a^** | | | |
| --- | --- | --- | --- |
| **Variable** | **Hazard Ratio** | **95% Confidence Interval** | **P-Value** |
| PPI use quintile  (Referent: Non-drug users) |  |  |  |
| 1^st^ (Lowest) | 0.97 | 0.97–0.97 | <0.001 |
| 2^nd^ | 0.94 | 0.94–0.94 | <0.001 |
| 3^rd^ | 0.93 | 0.93–0.93 | <0.001 |
| 4^th^ | 0.94 | 0.94–0.94 | <0.001 |
| 5^th^ (Highest) | 0.91 | 0.91–0.91 | <0.001 |
| H2-blocker use quintile  (Referent: Non-drug users) |  |  |  |
| 1^st^ (Lowest) | 1.01 | 1.01–1.01 | <0.001 |
| 2^nd^ | 0.95 | 0.95–0.95 | <0.001 |
| 3^rd^ | 0.92 | 0.92–0.92 | <0.001 |
| 4^th^ | 0.89 | 0.89–0.89 | <0.001 |
| 5^th^ (Highest) | 0.95 | 0.95–0.95 | <0.001 |

^a^Adjusted for age, operationalized as a categorical variable with each stratum representing an age quarter, mimicking Cox model results

H2: Histamine-2

PPI: Proton pump inhibitor

PSA: Prostate-specific antigen
