# Supplementary material for: Association of Cumulative Proton Pump Inhibitor Use with Prostate Cancer Risk and Outcomes: A Population-Based Cohort Study
Source: Cancer Res Commun. 2026 Jul 24;6(7):1769–76. doi: 10.1158/2767-9764.CRC-26-0098 (PMC13396002; doi:10.1158/2767-9764.CRC-26-0098)
Supplement: Supplementary Table 12 — Distribution of biopsy by drug use, based on unique patient data [file crc-26-0098_supplementary_table_12_suppst12.docx]

| **Supplementary Table 12. Distribution of biopsy by drug use, based on unique patient data** | | | | |
| --- | --- | --- | --- | --- |
| **Number of biopsies** | **PPI users**  **(n=168,890)** | **H2-blocker users**  **(n=42,939)** | **PPI and H2-blocker non-users**  **(n=375,167)** | **Total (n=559,425)** |
| 0 | 154,060 (91.2%) | 38,983 (90.8%) | 358,110 (95.5%) | 526,242 (94.1%) |
| 1 | 11,687 (6.9%) | 3,148 (7.3%) | 13,469 (3.6%) | 26,226 (4.7%) |
| 2 | 2,417 (1.4%) | 629 (1.5%) | 2,741 (0.7%) | 5,340 (1.0%) |
| ≥3 | 726 (0.4%) | 179 (0.4%) | 847 (0.2%) | 1,617 (0.3%) |

PPI: Proton pump inhibitor

H2: Histamine-2
