# Supplementary material for: Association of Cumulative Proton Pump Inhibitor Use with Prostate Cancer Risk and Outcomes: A Population-Based Cohort Study
Source: Cancer Res Commun. 2026 Jul 24;6(7):1769–76. doi: 10.1158/2767-9764.CRC-26-0098 (PMC13396002; doi:10.1158/2767-9764.CRC-26-0098)
Supplement: Supplementary Table 13 — Univariable logistic regression analysis for the outcome of prostate biopsy, using counting process data, by time-varying exposure of drug quintile [file crc-26-0098_supplementary_table_13_suppst13.docx]

| **Supplementary Table 13. Univariable logistic regression analysis for the outcome of prostate biopsy, using counting process data, by time-varying exposure of drug quintile** | | | |
| --- | --- | --- | --- |
| **Variable** | **Odds Ratio** | **95% Confidence Interval** | **P-Value** |
| PPI use quintile  (Referent: Non-drug users) |  |  |  |
| 1^st^ (Lowest) | 1.13 | 1.07–1.19 | <0.001 |
| 2^nd^ | 1.08 | 1.01–1.15 | 0.03 |
| 3^rd^ | 0.96 | 0.90–1.02 | 0.18 |
| 4^th^ | 0.83 | 0.78–0.89 | <0.001 |
| 5^th^ (Highest) | 0.61 | 0.57–0.67 | <0.001 |
| H2-blocker use quintile  (Referent: Non-drug users) |  |  |  |
| 1^st^ (Lowest) | 1.08 | 0.95–1.23 | 0.23 |
| 2^nd^ | 1.16 | 1.05–1.28 | 0.004 |
| 3^rd^ | 0.95 | 0.84–1.07 | 0.41 |
| 4^th^ | 0.96 | 0.85–1.08 | 0.52 |
| 5^th^ (Highest) | 0.71 | 0.62–0.81 | <0.001 |

H2: Histamine-2

PPI: Proton pump inhibitor
