# Supplementary material for: Association of Cumulative Proton Pump Inhibitor Use with Prostate Cancer Risk and Outcomes: A Population-Based Cohort Study
Source: Cancer Res Commun. 2026 Jul 24;6(7):1769–76. doi: 10.1158/2767-9764.CRC-26-0098 (PMC13396002; doi:10.1158/2767-9764.CRC-26-0098)
Supplement: Supplementary Table 14 — Multivariable logistic regression analysis for the outcome of prostate biopsy, using counting process data, by time-varying exposure of drug quintile [file crc-26-0098_supplementary_table_14_suppst14.docx]

| **Supplementary Table 14. Multivariable logistic regression analysis for the outcome of prostate biopsy, using counting process data, by time-varying exposure of drug quintile** | | | |
| --- | --- | --- | --- |
| **Variable** | **Odds Ratio** | **95% Confidence Interval** | **P-Value** |
| PPI use quintile  (Referent: Non-drug users) |  |  |  |
| 1^st^ (Lowest) | 1.16 | 1.11–1.23 | <0.001 |
| 2^nd^ | 1.14 | 1.07–1.22 | <0.001 |
| 3^rd^ | 1.04 | 0.98–1.10 | 0.25 |
| 4^th^ | 0.96 | 0.90–1.02 | 0.23 |
| 5^th^ (Highest) | 0.85 | 0.79–0.92 | <0.001 |
| H2-blocker use quintile  (Referent: Non-drug users) |  |  |  |
| 1^st^ (Lowest) | 1.17 | 1.04–1.33 | 0.01 |
| 2^nd^ | 1.24 | 1.13–1.37 | <0.001 |
| 3^rd^ | 1.04 | 0.93–1.17 | 0.47 |
| 4^th^ | 1.07 | 0.96–1.20 | 0.21 |
| 5^th^ (Highest) | 0.89 | 0.79–1.02 | 0.09 |
| Income quintile  (Referent: 5 [highest]) |  |  |  |
| 1 (lowest) | 0.81 | 0.78–0.85 | <0.001 |
| 2 | 0.91 | 0.87–0.95 | <0.001 |
| 3 | 0.92 | 0.88–0.96 | <0.001 |
| 4 | 0.95 | 0.92–0.99 | 0.014 |
| Rural | 0.83 | 0.80–0.86 | <0.001 |
| ADG (Referent score: 0) |  |  |  |
| 1-2 | 2.73 | 2.59–2.88 | <0.001 |
| 3-4 | 3.12 | 2.96–3.29 | <0.001 |
| 5-6 | 3.42 | 3.25–3.61 | <0.001 |
| 7+ | 3.55 | 3.37–3.75 | <0.001 |
| Asthma | 1.04 | 0.99–1.09 | 0.12 |
| COPD | 0.91 | 0.88–0.94 | <0.001 |
| CHF | 0.74 | 0.70–0.78 | <0.001 |
| Diabetes | 0.79 | 0.76–0.81 | <0.001 |
| Age | 0.60 | 0.59–0.62 | <0.001 |

ADG: Aggregated Diagnosis Groups

CHF: Congestive heart failure

COPD: Chronic obstructive pulmonary disease

H2: Histamine-2

PPI: Proton pump inhibitor

PSA: Prostate-specific antigen
