# Supplementary material for: Association of Cumulative Proton Pump Inhibitor Use with Prostate Cancer Risk and Outcomes: A Population-Based Cohort Study
Source: Cancer Res Commun. 2026 Jul 24;6(7):1769–76. doi: 10.1158/2767-9764.CRC-26-0098 (PMC13396002; doi:10.1158/2767-9764.CRC-26-0098)
Supplement: Supplementary Table 15 — Univariable logistic regression analysis (with complementary loglog link) for the outcome of the first ADT prescription or bilateral orchiectomy, using counting process data, by time-varying exposure of drug quintile [file crc-26-0098_supplementary_table_15_suppst15.docx]

| **Supplementary Table 15. Univariable logistic regression analysis (with complementary loglog link) for the outcome of the first ADT prescription or bilateral orchiectomy, using counting process data, by time-varying exposure of drug quintile^a^** | | | |
| --- | --- | --- | --- |
| **Variable** | **Hazard Ratio** | **95% Confidence Interval** | **P-Value** |
| PPI use quintile  (Referent: Non-drug users) |  |  |  |
| 1^st^ (Lowest) | 1.11 | 1.04–1.19 | 0.002 |
| 2^nd^ | 1.02 | 0.94–1.10 | 0.70 |
| 3^rd^ | 0.96 | 0.89–1.04 | 0.33 |
| 4^th^ | 0.95 | 0.88–1.02 | 0.16 |
| 5^th^ (Highest) | 0.91 | 0.85–0.99 | 0.02 |
| H2-blocker use quintile  (Referent: Non-drug users) |  |  |  |
| 1^st^ (Lowest) | 0.98 | 0.84–1.14 | 0.79 |
| 2^nd^ | 1.06 | 0.94–1.20 | 0.36 |
| 3^rd^ | 0.99 | 0.86–1.14 | 0.92 |
| 4^th^ | 0.99 | 0.86–1.14 | 0.86 |
| 5^th^ (Highest) | 0.92 | 0.80–1.06 | 0.27 |

^a^Adjusted for age, operationalized as a categorical variable with each stratum representing an age quarter, mimicking Cox model results

ADT: Androgen deprivation therapy

H2: Histamine-2

PPI: Proton pump inhibitor
