# Supplementary material for: Association of Cumulative Proton Pump Inhibitor Use with Prostate Cancer Risk and Outcomes: A Population-Based Cohort Study
Source: Cancer Res Commun. 2026 Jul 24;6(7):1769–76. doi: 10.1158/2767-9764.CRC-26-0098 (PMC13396002; doi:10.1158/2767-9764.CRC-26-0098)
Supplement: Supplementary Table 16 — Univariable logistic regression analysis (with complementary loglog link) for the outcome of the first PSA doubling time ≤6 months, using counting process data, by time-varying exposure of drug quintile [file crc-26-0098_supplementary_table_16_suppst16.docx]

| **Supplementary Table 16. Univariable logistic regression analysis (with complementary loglog link) for the outcome of the first PSA doubling time ≤6 months, using counting process data, by time-varying exposure of drug quintile^a^** | | | |
| --- | --- | --- | --- |
| **Variable** | **Hazard Ratio** | **95% Confidence Interval** | **P-Value** |
| PPI use quintile  (Referent: Non-drug users) |  |  |  |
| 1^st^ (Lowest) | 1.11 | 1.11–1.11 | <0.001 |
| 2^nd^ | 1.09 | 1.08–1.09 | <0.001 |
| 3^rd^ | 1.12 | 1.11–1.12 | <0.001 |
| 4^th^ | 1.03 | 1.03–1.03 | <0.001 |
| 5^th^ (Highest) | 1.08 | 1.08–1.08 | <0.001 |
| H2-blocker use quintile  (Referent: Non-drug users) |  |  |  |
| 1^st^ (Lowest) | 1.12 | 1.12–1.13 | <0.001 |
| 2^nd^ | 1.05 | 1.05–1.06 | <0.001 |
| 3^rd^ | 1.00 | 0.99–1.00 | <0.001 |
| 4^th^ | 1.01 | 1.01–1.02 | <0.001 |
| 5^th^ (Highest) | 1.01 | 1.01–1.02 | <0.001 |

^a^Adjusted for age, operationalized as a categorical variable with each stratum representing an age quarter, mimicking Cox model results

H2: Histamine-2

PPI: Proton pump inhibitor
