# Supplementary material for: Association of Cumulative Proton Pump Inhibitor Use with Prostate Cancer Risk and Outcomes: A Population-Based Cohort Study
Source: Cancer Res Commun. 2026 Jul 24;6(7):1769–76. doi: 10.1158/2767-9764.CRC-26-0098 (PMC13396002; doi:10.1158/2767-9764.CRC-26-0098)
Supplement: Supplementary Table 17 — Multivariable logistic regression analysis (with complementary loglog link) for the outcome of the first PSA doubling time ≤6 months, using counting process data, by time-varying exposure of drug quintile [file crc-26-0098_supplementary_table_17_suppst17.docx]

| **Supplementary Table 17. Multivariable logistic regression analysis (with complementary loglog link) for the outcome of the first PSA doubling time ≤6 months, using counting process data, by time-varying exposure of drug quintile^a^** | | | |
| --- | --- | --- | --- |
| **Variable** | **Hazard Ratio** | **95% Confidence Interval** | **P-Value** |
| PPI use quintile  (Referent: Non-drug users) |  |  |  |
| 1^st^ (Lowest) | 1.11 | 1.11–1.11 | <0.001 |
| 2^nd^ | 1.08 | 1.08–1.09 | <0.001 |
| 3^rd^ | 1.11 | 1.11–1.11 | <0.001 |
| 4^th^ | 1.02 | 1.02–1.02 | <0.001 |
| 5^th^ (Highest) | 1.06 | 1.06–1.06 | <0.001 |
| H2-blocker use quintile  (Referent: Non-drug users) |  |  |  |
| 1^st^ (Lowest) | 1.12 | 1.11–1.12 | <0.001 |
| 2^nd^ | 1.03 | 1.03–1.03 | <0.001 |
| 3^rd^ | 0.98 | 0.97–0.98 | <0.001 |
| 4^th^ | 0.99 | 0.99–0.99 | <0.001 |
| 5^th^ (Highest) | 0.99 | 0.98–0.99 | <0.001 |
| Income quintile  (Referent: 5 [highest]) |  |  |  |
| 1 (lowest) | 1.13 | 1.13–1.13 | <0.001 |
| 2 | 0.99 | 0.99–0.99 | <0.001 |
| 3 | 1.01 | 1.01–1.01 | <0.001 |
| 4 | 0.98 | 0.98–0.98 | <0.001 |
| Rural | 1.09 | 1.09–1.10 | <0.001 |
| ADG (Referent score: 0) |  |  |  |
| 1-2 | 0.70 | 0.70–0.70 | <0.001 |
| 3-4 | 0.63 | 0.62–0.63 | <0.001 |
| 5-6 | 0.62 | 0.62–0.62 | <0.001 |
| 7+ | 0.65 | 0.65–0.65 | <0.001 |
| Asthma | 1.05 | 1.05–1.05 | <0.001 |
| COPD | 1.02 | 1.02–1.02 | <0.001 |
| CHF | 1.21 | 1.20–1.21 | <0.001 |
| Diabetes | 1.05 | 1.05–1.05 | <0.001 |

^a^Adjusted for age, operationalized as a categorical variable with each stratum representing an age quarter, mimicking Cox model results

ADG: Aggregated Diagnosis Groups

CHF: Congestive heart failure

COPD: Chronic obstructive pulmonary disease

H2: Histamine-2

PPI: Proton pump inhibitor

PSA: Prostate-specific antigen
