# Supplementary material for: Association of Cumulative Proton Pump Inhibitor Use with Prostate Cancer Risk and Outcomes: A Population-Based Cohort Study
Source: Cancer Res Commun. 2026 Jul 24;6(7):1769–76. doi: 10.1158/2767-9764.CRC-26-0098 (PMC13396002; doi:10.1158/2767-9764.CRC-26-0098)
Supplement: Supplementary Table 18 — Univariable logistic regression analysis (with complementary loglog link) for the outcome of the any-cause death, using counting process data, by time-varying exposure of drug quintile [file crc-26-0098_supplementary_table_18_suppst18.docx]

| **Supplementary Table 18. Univariable logistic regression analysis (with complementary loglog link) for the outcome of any-cause death, using counting process data, by time-varying exposure of drug quintile^a^** | | | |
| --- | --- | --- | --- |
| **Variable** | **Hazard Ratio** | **95% Confidence Interval** | **P-Value** |
| PPI use quintile  (Referent: Non-drug users) |  |  |  |
| 1^st^ (Lowest) | 1.48 | 1.45–1.51 | <0.001 |
| 2^nd^ | 1.74 | 1.70–1.78 | <0.001 |
| 3^rd^ | 1.95 | 1.91–1.99 | <0.001 |
| 4^th^ | 2.01 | 1.97–2.05 | <0.001 |
| 5^th^ (Highest) | 1.81 | 1.78–1.85 | <0.001 |
| H2-blocker use quintile  (Referent: Non-drug users) |  |  |  |
| 1^st^ (Lowest) | 1.50 | 1.44–1.57 | <0.001 |
| 2^nd^ | 1.60 | 1.55–1.65 | <0.001 |
| 3^rd^ | 1.66 | 1.60–1.72 | <0.001 |
| 4^th^ | 1.69 | 1.63–1.75 | <0.001 |
| 5^th^ (Highest) | 1.63 | 1.57–1.68 | <0.001 |
| Eye drop use quintile  (Referent: Non-drug users) |  |  |  |
| 1^st^ (Lowest) | 1.00 | 0.96–1.05 | 0.82 |
| 2^nd^ | 1.08 | 1.04–1.13 | 0.002 |
| 3^rd^ | 1.10 | 1.06–1.16 | 0.042 |
| 4^th^ | 1.06 | 1.02–1.11 | 0.25 |
| 5^th^ (Highest) | 1.02 | 0.98–1.07 | 0.005 |

^a^Adjusted for age, operationalized as a categorical variable with each stratum representing an age quarter, mimicking Cox model results

H2: Histamine-2

PPI: Proton pump inhibitor
