# Supplementary material for: Association of Cumulative Proton Pump Inhibitor Use with Prostate Cancer Risk and Outcomes: A Population-Based Cohort Study
Source: Cancer Res Commun. 2026 Jul 24;6(7):1769–76. doi: 10.1158/2767-9764.CRC-26-0098 (PMC13396002; doi:10.1158/2767-9764.CRC-26-0098)
Supplement: Supplementary Table 19 — Multivariable logistic regression analysis (with complementary loglog link) for the outcome of the any-cause death, using counting process data, by time-varying exposure of drug quintile [file crc-26-0098_supplementary_table_19_suppst19.docx]

| **Supplementary Table 19.** **Multivariable logistic regression analysis (with complementary loglog link) for the outcome of the any-cause death, using counting process data, by time-varying exposure of drug quintile*** | | | |
| --- | --- | --- | --- |
| **Variable** | Hazard Ratio | 95% Confidence Interval | P-Value |
| PPI use quintile  (Referent: Non-drug users) |  |  |  |
| 1^st^ (Lowest) | 1.13 | 1.11–1.16 | <0.001 |
| 2^nd^ | 1.26 | 1.24–1.29 | <0.001 |
| 3^rd^ | 1.36 | 1.33–1.39 | <0.001 |
| 4^th^ | 1.31 | 1.29–1.34 | <0.001 |
| 5^th^ (Highest) | 1.11 | 1.09–1.14 | <0.001 |
| H2-blocker use quintile  (Referent: Non-drug users) |  |  |  |
| 1^st^ (Lowest) | 1.13 | 1.09–1.18 | <0.001 |
| 2^nd^ | 1.18 | 1.14–1.22 | <0.001 |
| 3^rd^ | 1.20 | 1.16–1.24 | <0.001 |
| 4^th^ | 1.20 | 1.16–1.24 | <0.001 |
| 5^th^ (Highest) | 1.10 | 1.06–1.14 | <0.001 |
| Income quintile  (Referent: 5 [highest]) |  |  |  |
| 1 (lowest) | 1.36 | 1.34–1.39 | <0.001 |
| 2 | 1.22 | 1.20–1.24 | <0.001 |
| 3 | 1.14 | 1.12–1.16 | <0.001 |
| 4 | 1.07 | 1.05–1.09 | <0.001 |
| Rural | 1.32 | 1.29–1.34 | <0.001 |
| ADG (Referent score: 0) |  |  |  |
| 1-2 | 2.37 | 2.33–2.42 | <0.001 |
| 3-4 | 2.18 | 2.14–2.23 | <0.001 |
| 5-6 | 2.19 | 2.14–2.23 | <0.001 |
| 7+ | 2.45 | 2.40–2.49 | <0.001 |
| Asthma | 0.86 | 0.84–0.87 | <0.001 |
| COPD | 1.79 | 1.77–1.81 | <0.001 |
| CHF | 2.29 | 2.27–2.32 | <0.001 |
| Diabetes | 1.21 | 1.20–1.22 | <0.001 |

*Adjusted for age, operationalized as a categorical variable with each stratum representing an age quarter, mimicking Cox model results

ADT: Androgen deprivation therapy

ADG: Aggregated Diagnosis Groups

CHF: Congestive heart failure

COPD: Chronic obstructive pulmonary disease

H2: Histamine-2

PPI: Proton pump inhibitor

PSA: Prostate-specific antigen
