# Supplementary material for: Association of Cumulative Proton Pump Inhibitor Use with Prostate Cancer Risk and Outcomes: A Population-Based Cohort Study
Source: Cancer Res Commun. 2026 Jul 24;6(7):1769–76. doi: 10.1158/2767-9764.CRC-26-0098 (PMC13396002; doi:10.1158/2767-9764.CRC-26-0098)
Supplement: Supplementary Table 20 — Summary of patients, outcome events and records [file crc-26-0098_supplementary_table_20_suppst20.docx]

| **Supplementary Table 20. Summary of patients, outcome events and records** | | |
| --- | --- | --- |
| **Unique patients** | **No. events** | **Records** |
| PSA ≥4 ng/ml in patients with ≥1 PSA test | | |
| 277,991 | 71,621 | 2,083,722 |
| PSA velocity >0.75 ng/ml/year with ≥2 PSA tests | | |
| 197,914 | 62,920 | 1,672,679 |
| Prostate biopsy | | |
| 559,425 | 33,183 | 5,414,176 |
| Prostate cancer diagnosis in overall cohort | | |
| 559,425 | 46,799 | 5,371,525 |
| Clinically significant prostate cancer diagnosis (i.e., Gleason Score ≥7) in overall cohort | | |
| 559,425 | 8,701 | 5,613,236 |
| High-grade prostate cancer diagnosis (i.e., Gleason Score ≥8) in overall cohort | | |
| 559,425 | 3,408 | 5,643,322 |
| ADT or bilateral orchiectomy | | |
| 559,425 | 15,827 | 5,583,429 |
| PSA doubling time ≤6 months | | |
| 197,914 | 19,302 | 1,830,493 |
| Any-cause death | | |
| 559,425 | 155,487 | 5,658,595 |

ADT: Androgen deprivation therapy

PSA: Prostate-specific antigen
