# Supplementary material for: Association of Cumulative Proton Pump Inhibitor Use with Prostate Cancer Risk and Outcomes: A Population-Based Cohort Study
Source: Cancer Res Commun. 2026 Jul 24;6(7):1769–76. doi: 10.1158/2767-9764.CRC-26-0098 (PMC13396002; doi:10.1158/2767-9764.CRC-26-0098)
Supplement: Supplementary Table 21 — Person-time rates of study outcomes, by exposure, based on the counting process data [file crc-26-0098_supplementary_table_21_suppst21.docx]

| **Supplementary Table 21. Person-time rates and median follow-up time of study outcomes, by exposure, based on the individual data** | | | | | |
| --- | --- | --- | --- | --- | --- |
|  | **Both PPI and H2 blockes** | **PPI only** | **H2 blocker only** | **Non-drug use** | **Overall** |
| PSA ≥4 ng/ml in patients with ≥1 PSA test | | | | | |
| Sample size | 17,730 | 92,197 | 7,927 | 160,137 | 277,991 |
| Number of events | 5,036 | 24,909 | 2,316 | 39,360 | 71,621 |
| Person years | 157,014 | 737,099 | 61,573 | 987,975 | 1,943,661 |
| Median follow-up, years | 8.6 | 7.8 | 7.5 | 5.7 | 6.7 |
| Rate per 100 person-years (95% CI) | 3.2 (3.1-3.3) | 3.4 (3.3-3.4) | 3.8 (3.6-3.9) | 4.0 (3.9-4.0) | 3.7 (3.7-3.7) |
| PSA velocity >0.75 ng/ml/year with ≥2 PSA tests | | | | | |
| Sample size | 13,362 | 68,962 | 5,744 | 109,846 | 197,914 |
| Number of events | 4,597 | 22,737 | 2,019 | 33,567 | 62,920 |
| Person years | 128,351 | 604,014 | 49,523 | 792,075 | 1,573,963 |
| Median follow-up, years | 9.5 | 8.6 | 8.4 | 6.8 | 7.7 |
| Rate per 100 person-years (95% CI) | 3.6 (3.5-3.7) | 3.8 (3.7-3.8) | 4.1 (3.9-4.3) | 4.2 (4.2-4.3) | 4.0 (4.0-4.0) |
| Prostate biopsy in overall cohort | | | | | |
| Sample size | 27,571 | 141,319 | 15,368 | 375,167 | 559,425 |
| Number of events | 2,660 | 12,170 | 1,296 | 17,057 | 33,183 |
| Person years | 314,790 | 1,489,856 | 141,668 | 3,172,759 | 5,119,073 |
| Median follow-up, years | 12.1 | 10.7 | 8.8 | 7.4 | 8.5 |
| Rate per 100 person-years (95% CI) | 0.8 (0.8-0.9) | 0.8 (0.8-0.8) | 0.9 (0.9-1.0) | 0.5 (0.5-0.5) | 0.6 (0.6-0.7) |
| Prostate cancer diagnosis in overall cohort | | | | | |
| Sample size | 27,571 | 141,319 | 15,368 | 375,167 | 559,425 |
| Number of events | 3,884 | 17,264 | 1,919 | 23,732 | 46,799 |
| Person years | 310,552 | 1,472,573 | 139,636 | 3,154,160 | 5,076,922 |
| Median follow-up, years | 11.9 | 10.5 | 8.6 | 7.3 | 8.4 |
| Rate per 100 person-years (95% CI) | 1.3 (1.2-1.3) | 1.2 (1.2-1.2) | 1.4 (1.3-1.4) | 0.8 (0.7-0.8) | 0.9 (0.9-0.9) |
| Clinically significant prostate cancer diagnosis (i.e., Gleason Score ≥7) in overall cohort | | | | | |
| Sample size | 27,571 | 141,319 | 15,368 | 375,167 | 559,425 |
| Number of events | 675 | 3,308 | 346 | 4,372 | 8,701 |
| Person years | 334,006 | 1,568,687 | 149,069 | 3,265,475 | 5,317,238 |
| Median follow-up, years | 12.9 | 11.4 | 9.5 | 7.7 | 9.1 |
| Rate per 100 person-years (95% CI) | 0.2 (0.2-0.2) | 0.2 (0.2-0.2) | 0.2 (0.2-0.3) | 0.1 (0.1-0.1) | 0.2 (0.2-0.2) |
| High-grade prostate cancer diagnosis (i.e., Gleason Score ≥8) in overall cohort | | | | | |
| Sample size | 27,571 | 141,319 | 15,368 | 375,167 | 559,425 |
| Number of events | 308 | 1,330 | 149 | 1,621 | 3,408 |
| Person years | 336,197 | 1,580,094 | 150,190 | 3,280,745 | 5,347,226 |
| Median follow-up, years | 13.1 | 11.5 | 9.6 | 7.7 | 9.1 |
| Rate per 100 person-years (95% CI) | 0.1 (0.1-0.1) | 0.1 (0.1-0.1) | 0.1 (0.1-0.1) | 0.0 (0.0-0.1) | 0.1 (0.1-0.1) |
| ADT or bilateral orchiectomy | | | | | |
| Sample size | 27,571 | 141,319 | 15,368 | 375,167 | 559,425 |
| Number of events | 1,389 | 6,042 | 747 | 7,649 | 15,827 |
| Person years | 330,115 | 1,555,740 | 147,395 | 3,254,734 | 5,287,985 |
| Median follow-up, years | 12.7 | 11.3 | 9.4 | 7.6 | 9.0 |
| Rate per 100 person-years (95% CI) | 0.4 (0.4-0.4) | 0.4 (0.4-0.4) | 0.5 (0.5-0.5) | 0.2 (0.2-0.2) | 0.3 (0.3-0.3) |
| PSA doubling time ≤6 months | | | | | |
| Sample size | 13,362 | 68,962 | 5,744 | 109,846 | 197,914 |
| Number of events | 1,685 | 7,496 | 709 | 9,412 | 19,302 |
| Person years | 140,227 | 662,763 | 54,595 | 874,194 | 1,731,778 |
| Median follow-up, years | 10.6 | 9.6 | 9.3 | 7.5 | 8.6 |
| Rate per 100 person-years (95% CI) | 1.2 (1.1-1.3) | 1.1 (1.1-1.2) | 1.3 (1.2-1.4) | 1.1 (1.1-1.1) | 1.1 (1.1-1.1) |
| Any-cause death | | | | | |
| Sample size | 27,571 | 141,319 | 15,368 | 20,205 | 354,962 |
| Number of events | 11,269 | 49,796 | 7,928 | 4,850 | 81,644 |
| Person years | 337,612 | 1,586,063 | 150,834 | 233,412 | 3,054,493 |
| Median follow-up, years | 13.1 | 11.5 | 9.7 | 12.0 | 7.5 |
| Rate per 100 person-years (95% CI) | 3.3 (3.3-3.4) | 3.1 (3.1-3.2) | 5.3 (5.1-5.4) | 2.1 (2.0-2.1) | 2.7 (2.7-2.7) |

ADT: Androgen deprivation therapy

CI: Confidence Interval

H2: Histamine-2

PPI: Proton pump inhibitor

PSA: Prostate-specific antigen
