# Supplementary material for: Association of Cumulative Proton Pump Inhibitor Use with Prostate Cancer Risk and Outcomes: A Population-Based Cohort Study
Source: Cancer Res Commun. 2026 Jul 24;6(7):1769–76. doi: 10.1158/2767-9764.CRC-26-0098 (PMC13396002; doi:10.1158/2767-9764.CRC-26-0098)
Supplement: Supplementary Table 22 — Drug dosage by quintile for the study outcomes, based on the counting process data [file crc-26-0098_supplementary_table_22_suppst22.docx]

| **Supplementary Table 22. Drug dosage by quintile for the study outcomes, based on the counting process data** | | | |
| --- | --- | --- | --- |
| **Drug Quintile** | **Value** | **PPI (grams)** | **H2-Blockers (grams)** |
| PSA ≥4 ng/ml in patients with ≥1 PSA test | | | |
| 1 (Lowest) | No. records | N=105,155 | N=10,983 |
|  | Median (IQR) | 1200 (600-1200) | 22386 (12792-23985) |
|  | Min – Max | 20 - 1200 | 600 - 46371 |
| 2 | No. records | N=61,118 | N=13,845 |
|  | Median (IQR) | 2400 (2240-3000) | 47970 (47970-47970) |
|  | Min – Max | 1202 - 3600 | 47970 - 47970 |
| 3 | No. records | N=77,417 | N=10,359 |
|  | Median (IQR) | 6603 (4800-8760) | 95940 (92742-108732) |
|  | Min – Max | 3602 - 11993 | 48000 - 143970 |
| 4 | No. records | N=83,081 | N=10,219 |
|  | Median (IQR) | 21161 (15600-28274) | 287820 (191880-383760) |
|  | Min – Max | 12000 - 37845 | 144000 - 527970 |
| 5 (Highest) | No. records | N=81,686 | N=10,637 |
|  | Median (IQR) | 68400 (50400-98360) | 1327170 (831480-2318550) |
|  | Min – Max | 37847 - 455769 | 528000 - 13549926 |
| PSA velocity >0.75 ng/ml/year with ≥2 PSA tests | | | |
| 1 (Lowest) | No. records | N=88,871 | N=9,139 |
|  | Median (IQR) | 1200 (600-1200) | 22386 (12600-23985) |
|  | Min – Max | 20 - 1200 | 1200 - 46371 |
| 2 | No. records | N=51,823 | N=11,232 |
|  | Median (IQR) | 2400 (2240-3003) | 47970 (47970-47970) |
|  | Min – Max | 1202 - 3600 | 47970 - 47970 |
| 3 | No. records | N=65,453 | N=8,475 |
|  | Median (IQR) | 6600 (4800-8680) | 95940 (89544-111930) |
|  | Min – Max | 3602 - 11993 | 48000 - 143910 |
| 4 | No. records | N=70,770 | N=8,346 |
|  | Median (IQR) | 21523 (15600-28800) | 287820 (191880-383760) |
|  | Min – Max | 12000 - 38400 | 143940 - 527670 |
| 5 (Highest) | No. records | N=69,110 | N=8,445 |
|  | Median (IQR) | 69600 (51520-99600) | 1333566 (831480-2350530) |
|  | Min – Max | 38403 - 455769 | 527730 - 13591500 |
| Prostate biopsy in overall cohort | | | |
| 1 (Lowest) | No. records | N=245,478 | N=25,404 |
|  | Median (IQR) | 1200 (600-1200) | 22386 (11193-23985) |
|  | Min – Max | 20 - 1200 | 600 - 46371 |
| 2 | No. records | N=170,465 | N=32,692 |
|  | Median (IQR) | 2403 (2361-3600) | 47970 (47970-47970) |
|  | Min – Max | 1201 - 4780 | 47970 - 48000 |
| 3 | No. records | N=212,089 | N=24,608 |
|  | Median (IQR) | 8400 (6000-11120) | 95940 (95940-143910) |
|  | Min – Max | 4800 - 15200 | 49569 - 186000 |
| 4 | No. records | N=208,909 | N=24,182 |
|  | Median (IQR) | 27440 (20400-36045) | 319800 (223860-447720) |
|  | Min – Max | 15201 - 47960 | 188682 - 639600 |
| 5 (Highest) | No. records | N=209,225 | N=24,668 |
|  | Median (IQR) | 84000 (62920-118308) | 1567020 (997776-2718300) |
|  | Min – Max | 47961 - 511483 | 639970 - 18484440 |
| Prostate cancer diagnosis in overall cohort | | | |
| 1 (Lowest) | No. records | N=241,898 | N=25,057 |
|  | Median (IQR) | 1200 (600-1200) | 22386 (11193-23985) |
|  | Min – Max | 20 - 1200 | 600 - 46371 |
| 2 | No. records | N=167,726 | N=31,999 |
|  | Median (IQR) | 2403 (2360-3600) | 47970 (47970-47970) |
|  | Min – Max | 1201 - 4780 | 47970 - 48000 |
| 3 | No. records | N=206,389 | N=24,184 |
|  | Median (IQR) | 8365 (6000-10812) | 95940 (95940-143910) |
|  | Min – Max | 4800 - 14964 | 49569 - 186000 |
| 4 | No. records | N=205,326 | N=23,614 |
|  | Median (IQR) | 27000 (20240-36000) | 311805 (217464-431730) |
|  | Min – Max | 14964 - 47295 | 188682 - 638001 |
| 5 (Highest) | No. records | N=205,334 | N=24,364 |
|  | Median (IQR) | 83304 (62400-117466) | 1536000 (973791-2686320) |
|  | Min – Max | 47296 - 511483 | 639600 - 18484440 |
| Clinically significant prostate cancer diagnosis (i.e., Gleason Score ≥7) in overall cohort | | | |
| 1 (Lowest) | No. records | N=260,958 | N=27,213 |
|  | Median (IQR) | 1200 (600-1200) | 22386 (11193-23985) |
|  | Min – Max | 20 - 1200 | 600 - 46371 |
| 2 | No. records | N=180,966 | N=34,578 |
|  | Median (IQR) | 2403 (2360-3600) | 47970 (47970-47970) |
|  | Min – Max | 1201 - 4780 | 47970 - 48000 |
| 3 | No. records | N=227,125 | N=26,152 |
|  | Median (IQR) | 8400 (6000-11200) | 95940 (95940-143910) |
|  | Min – Max | 4800 - 15540 | 49569 - 186000 |
| 4 | No. records | N=223,037 | N=25,518 |
|  | Median (IQR) | 27600 (20520-36640) | 319800 (222291-436527) |
|  | Min – Max | 15560 - 48200 | 188682 - 639600 |
| 5 (Highest) | No. records | N=222,994 | N=25,961 |
|  | Median (IQR) | 84880 (63600-119400) | 1583010 (1007370-2734290) |
|  | Min – Max | 48201 - 511483 | 639970 - 18484440 |
| High-grade prostate cancer diagnosis (i.e., Gleason Score ≥8) in overall cohort | | | |
| 1 (Lowest) | No. records | N=263,496 | N=27,424 |
|  | Median (IQR) | 1200 (600-1200) | 22386 (11193-23985) |
|  | Min – Max | 20 - 1200 | 600 - 46371 |
| 2 | No. records | N=182,810 | N=35,003 |
|  | Median (IQR) | 2403 (2360-3600) | 47970 (47970-47970) |
|  | Min – Max | 1201 - 4780 | 47970 - 48000 |
| 3 | No. records | N=229,400 | N=26,383 |
|  | Median (IQR) | 8400 (6000-11200) | 95940 (95940-143910) |
|  | Min – Max | 4800 - 15580 | 49569 - 185484 |
| 4 | No. records | N=225,461 | N=25,449 |
|  | Median (IQR) | 27600 (20560-36760) | 311805 (215865-431730) |
|  | Min – Max | 15600 - 48390 | 186000 - 638001 |
| 5 (Highest) | No. records | N=225,433 | N=26,376 |
|  | Median (IQR) | 85080 (63761-119560) | 1567020 (983385-2718300) |
|  | Min – Max | 48400 - 511483 | 639600 - 18484440 |
| ADT or bilateral orchiectomy | | | |
| 1 (Lowest) | No. records | N=258,653 | N=26,804 |
|  | Median (IQR) | 1200 (600-1200) | 22386 (11193-23985) |
|  | Min – Max | 20 - 1200 | 600 - 46371 |
| 2 | No. records | N=179,319 | N=34,276 |
|  | Median (IQR) | 2403 (2360-3600) | 47970 (47970-47970) |
|  | Min – Max | 1201 - 4780 | 47970 - 48000 |
| 3 | No. records | N=223,730 | N=25,847 |
|  | Median (IQR) | 8400 (6000-11200) | 95940 (95940-143910) |
|  | Min – Max | 4800 - 15361 | 49569 - 186000 |
| 4 | No. records | N=220,568 | N=25,018 |
|  | Median (IQR) | 27600 (20400-36400) | 310206 (215865-431730) |
|  | Min – Max | 15361 - 48012 | 188682 - 638001 |
| 5 (Highest) | No. records | N=220,566 | N=25,898 |
|  | Median (IQR) | 84600 (63600-119000) | 1567020 (981786-2718300) |
|  | Min – Max | 48013 - 511483 | 639600 - 18484440 |
| PSA doubling time ≤6 months | | | |
| 1 (Lowest) | No. records | N=101,800 | N=10,277 |
|  | Median (IQR) | 1200 (600-1200) | 22386 (11193-23985) |
|  | Min – Max | 20 - 1200 | 1200 - 46371 |
| 2 | No. records | N=59,335 | N=12,619 |
|  | Median (IQR) | 2400 (2240-3003) | 47970 (47970-47970) |
|  | Min – Max | 1202 - 3600 | 47970 - 47970 |
| 3 | No. records | N=79,316 | N=9,312 |
|  | Median (IQR) | 7200 (4806-9200) | 95940 (89544-111930) |
|  | Min – Max | 3602 - 12380 | 48000 - 143910 |
| 4 | No. records | N=80,120 | N=9,159 |
|  | Median (IQR) | 22800 (16800-30240) | 272000 (191880-383760) |
|  | Min – Max | 12400 - 40800 | 143940 - 527970 |
| 5 (Highest) | No. records | N=80,180 | N=9,505 |
|  | Median (IQR) | 73000 (54000-104000) | 1375140 (847470-2446470) |
|  | Min – Max | 40800 - 455769 | 528000 - 13591500 |
| Any-cause death | | | |
| 1 (Lowest) | No. records | N=27,568 | N=34,317 |
|  | Median (IQR) | 22386 (11193-23985) | 7 (5-10) |
|  | Min – Max | 600 - 46371 | 1 - 14 |
| 2 | No. records | N=35,219 | N=36,717 |
|  | Median (IQR) | 47970 (47970-47970) | 30 (24-54) |
|  | Min – Max | 47970 - 48000 | 15 - 80 |
| 3 | No. records | N=26,489 | N=36,650 |
|  | Median (IQR) | 95940 (95940-143910) | 180 (120-245) |
|  | Min – Max | 49569 - 183885 | 81 - 333 |
| 4 | No. records | N=25,571 | N=35,177 |
|  | Median (IQR) | 310206 (215865-431730) | 570 (440-740) |
|  | Min – Max | 185484 - 638001 | 334 - 960 |
| 5 (Highest) | No. records | N=26,424 | N=31,417 |
|  | Median (IQR) | 1567020 (983385-2718300) | 1640 (1240-2370) |
|  | Min – Max | 639600 - 18484440 | 961 - 15732 |

ADT: Androgen deprivation therapy

H2: Histamine-2

IQR: Interquartile range

PPI: Proton pump inhibitor

PSA: Prostate-specific antigen
