# Supplementary figures and images for: Association of Cumulative Proton Pump Inhibitor Use with Prostate Cancer Risk and Outcomes: A Population-Based Cohort Study
Source: Cancer Res Commun. 2026 Jul 24;6(7):1769–76. doi: 10.1158/2767-9764.CRC-26-0098 (PMC13396002; doi:10.1158/2767-9764.CRC-26-0098)

**Supplementary Figure 1. Study flow chart**

***
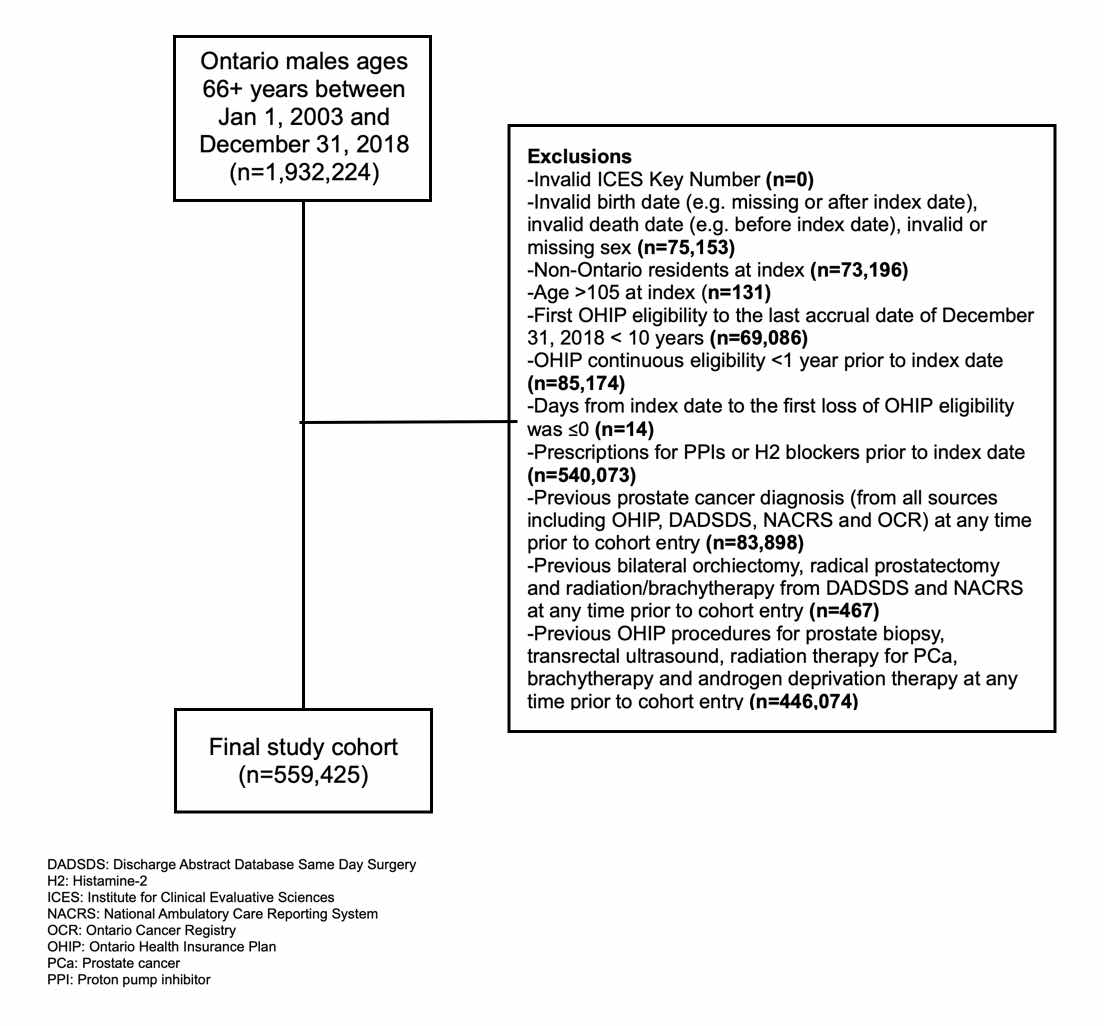
***

Supplement: Supplementary Figure 1 — Study flow chart [file crc-26-0098_supplementary_figure_1_suppsf1.docx]
